# Polycarboxybetaine-Based Hydrogels for the Capture and Release of Circulating Tumor Cells

**DOI:** 10.3390/gels8070391

**Published:** 2022-06-21

**Authors:** Hsiu-Wen Chien, Jen-Chia Wu, Ying-Chih Chang, Wei-Bor Tsai

**Affiliations:** 1 Department of Chemical and Material Engineering, National Kaohsiung University of Science and Technology, Kaohsiung 807, Taiwan; hsiu-wen.chien@nkust.edu.tw; 2 Genomics Research Center, Academia Sinica, Taipei 115, Taiwan; jenchiawu@gmail.com; 3 Department of Chemical Engineering, Stanford University, Stanford, CA 94305, USA; 4 Department of Chemical Engineering, National Taiwan University, Taipei 106, Taiwan

**Keywords:** non-fouling, polycarboxybetaine, hydrogel, circulating tumor cells, anti-EpCAM

## Abstract

Circulating tumor cells (CTCs) are indicators for the detection, diagnosis, and monitoring of cancers and offer biological information for the development of personalized medicine. Techniques for the specific capture and non-destructive release of CTCs from millions of blood cells remain highly desirable. Here, we present a CTC capture-and-release system using a disulfide-containing poly(carboxybetaine methacrylate) (pCB) hydrogel. The non-fouling characteristic of pCB prevents unwanted, nonspecific cell binding, while the carboxyl functionality of pCB is used for the conjugation of anti-epithelial cell adhesion molecule (anti-EpCAM) antibodies for the capture of CTCs. The results demonstrated that the anti-EpCAM-conjugated pCB hydrogel captured HCT116 cells from blood, and the capture ratio reached 45%. Furthermore, the captured HCT116 cells were released within 30 min from the dissolution of the pCB hydrogel by adding cysteine, which breaks the disulfide bonds of the crosslinkers. The cells released were viable and able to grow. Our system has potential in the development of a device for CTC diagnosis.

## 1. Introduction

Circulating tumor cells (CTCs) in the blood serve as biomarkers for the diagnosis, prognosis, and monitoring of metastatic cancers [1,2]. However, the detection and isolation of CTCs is not an easy task due to the extremely sparse population of CTCs in the blood, with as few as one cell per 10^9^ blood cells in patients with metastatic cancer [1]. Therefore, the enrichment or concentration of CTCs in blood samples is necessary for the isolation and diagnosis of CTCs.

In order to distinguish rare CTCs from other blood cells, the utilization of highly overexpressed surfaces markers of CTCs is a common approach. One of the common biomarkers for the identification of CTCs is the epithelial cell adhesion molecule (EpCAM), a 30–40 kDa glycosylated type I membrane protein [3,4], which is frequently overexpressed in cancer cell membranes, but is absent from hematologic cells, making the protein a good candidate for use in the efficient isolation of CTCs from blood. Therefore, anti-EpCAM antibodies have been utilized in devices for the detection and capture of CTCs [5,6,7,8,9].

Two critical issues determine the success of the ‘CTC chips’ for the detection and enrichment of CTC. One is the elimination of nonspecific binding of nontarget cells to a platform via physiological sorption, a source of impurity of isolated CTCs. For example, in some ‘CTC catch’ microchips, the identified CTCs exhibited an impurity of approximately 50% [5]. The other issue is the capability to release captured CTCs non-destructively. A non-fouling surface could prevent the nonspecific capture of nontarget cells, while a facile cell-detachment mechanism is needed for cell release. To this end, Li et al. designed an aptamer-functionalized polyacrylamide hydrogel, in which the polyacrylamide hydrogel serves as a low-fouling platform to resist nonspecific cell attachment, while cancer cells bind to the aptamer domains, and are later released through a break in the aptamer by a restriction endonuclease [10]. Another example is that Wu et al. designed a microfluidic device coated with a non-fouling phosphocholine bilayer that is conjugated with antibodies [6]. The conjugation of anti-EpCAM antibodies enables the phosphocholine substrate to bind CTCs, which are released by the addition of EDTA to disrupt the binding between cells and antibodies.

Phosphocholine, known for its antifouling ability [11], belongs to a family of zwitterionic molecules, including phosphobetaine, sulfobetaine, and carboxybetaine, which are electrically neutral molecules that contain positively and negatively charged motifs. The high binding capacity of zwitterionic molecules to water molecules due to electrostatic attraction makes them excellent non-fouling materials to prevent the nonspecific protein adsorption, cell adhesion, and bacterial attachment [12,13,14,15,16,17]. It is known that the surface coating of a poly(carboxybetaine methacrylate) (pCB) hydrogel layer on an implant could prevent the implant-initiated foreign body reaction [18]. Among the zwitterionic materials, carboxybetaine has the unique advantage in that it contains a carboxyl group that could be used for the conjugation of biomolecules via a carbodiimide reaction for the specific mediation of biological interactions [19,20,21,22].

**Figure 1:**
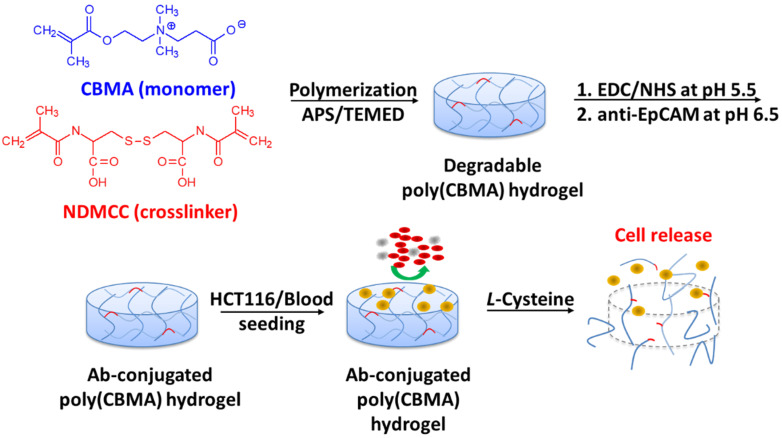
Schematic illustrations of preparing degradable anti-EpCAM-conjugated pCB hydrogels.

Previously, we developed a pCB hydrogel that resists cell attachment [23,24,25]. The conjugation of biomolecules on the pCB hydrogel could support specific cell interactions [24,25]. When the hydrogel contains disulfide-contained crosslinkers, the hydrogel could be dissolved using cysteine, a natural amino acid, by breaking the disulfide bonds. Our previous study demonstrated that cells were able to encapsulate in such a pCB hydrogel, and the cells could be recovered without damage by adding cysteine [25]. The present study aims to apply the pCB hydrogel for the capture and harvesting of CTCs. The pCB hydrogel was conjugated with anti-EpCAM antibodies for the capture of tumor cells, and cell release was achieved by adding cysteine (Figure 1). The capture efficacy and viability of the released tumor cells were evaluated.

## 2. Results and Discussion

### 2.1. The Resistance of pCB Hydrogel to Cell Adhesion

**Figure 2:**
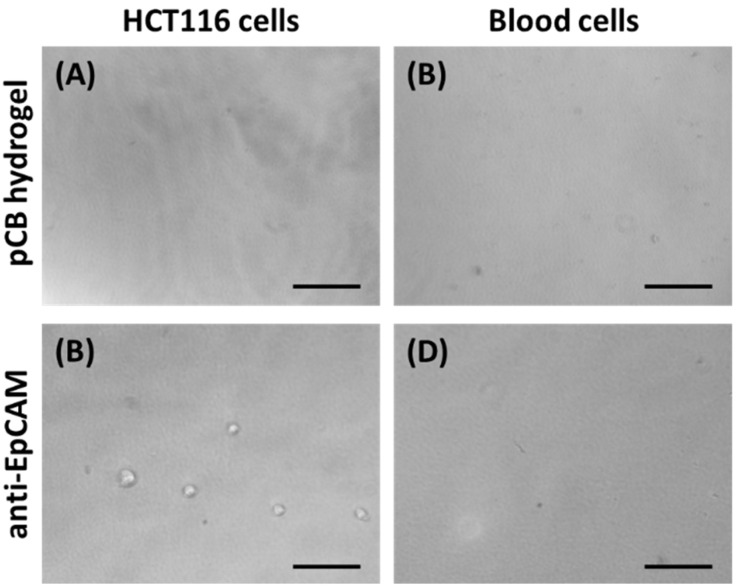
Phase-contrast microscopic images of HCT116 cells (**A,C**) and diluted blood (**B**,**D**) were incubated on pCB or anti-EpCAM-conjugated hydrogels for 4 h. Scale bar = 100 μm.

CTCs are tumor cells disseminated from primary tumors that subsequently travel through the blood circulation to distant organs. Thus, the prevention of billions of blood cell adhesion is an important criterion when building a suitable device. The pCB hydrogel is known to resist cell adhesion [26,27]. In this study, the HCT116 cells did not adhere to the pCB hydrogel after 4 h of culture (Figure 2A). Even after 3 days of culture, HCT116 cells still did not adhere to the pCB hydrogel. Furthermore, blood cells did not adhere to the surfaces of the pCB hydrogel (Figure 2B).

After the conjugation of anti-EpCAM to the surfaces of the pCB hydrogel, HCT116 cells were allowed to adhere to the hydrogel (Figure 2C). However, the adherent resistance of the anti-EpCAM-conjugated pCB hydrogel surfaces to blood cells remained (Figure 2D). The anti-EpCAM-conjugated pCB hydrogel resulted in the specific binding of CTCs while maintaining resistance to the nonspecific blood cell adhesion. The results indicate the potential of the pCB hydrogel as a non-fouling platform for the covalent conjugation of anti-EpCAM antibodies for the capture of CTCs.

### 2.2. Cell Capture on the Anti-EpCAM Antibody-Conjugated pCB Hydrogel

**Figure 3:**
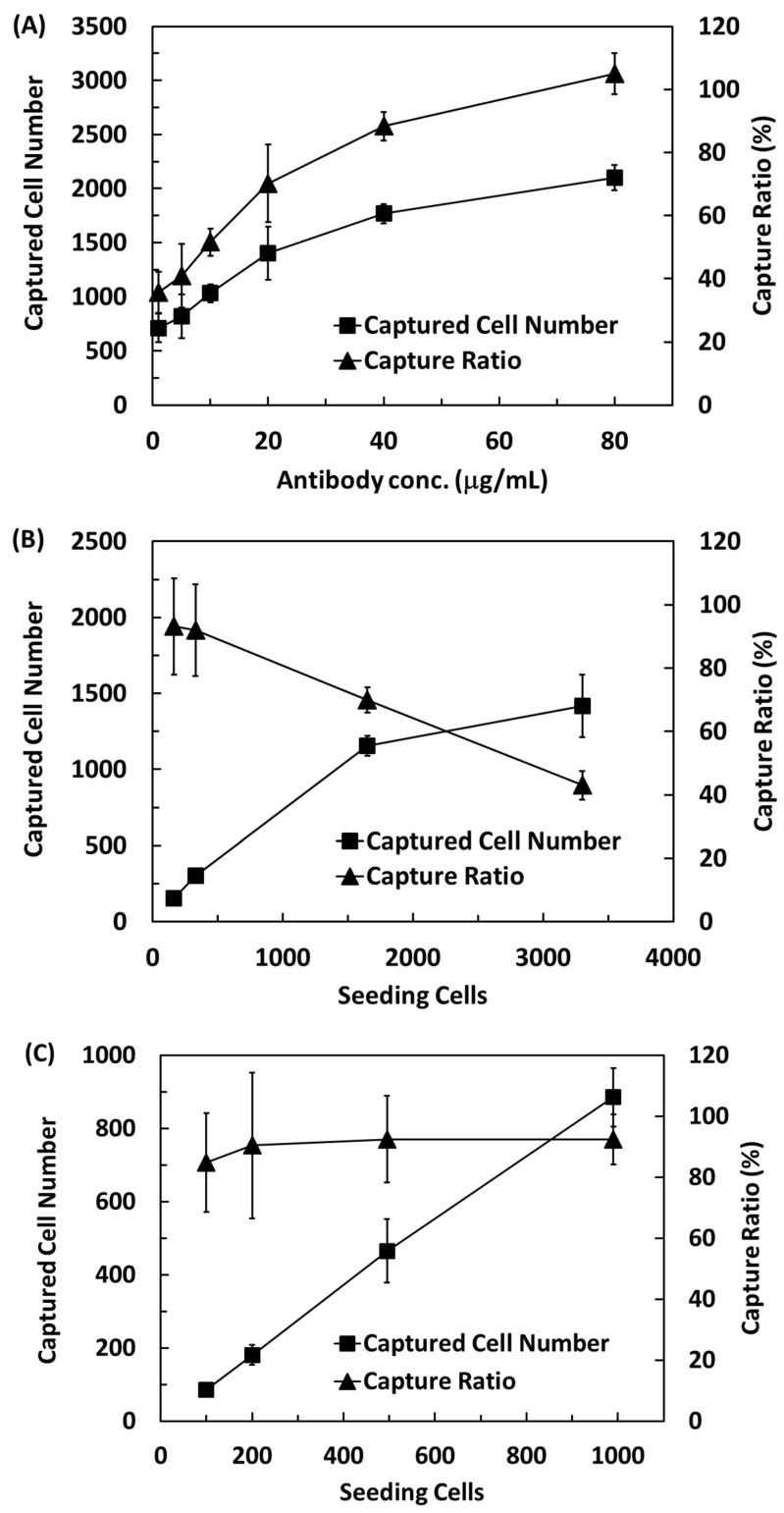
(**A**) The number of captured HCT116 cells and capture ratio as a function of anti-EpCAM conjugation concentration under seeding of 2200 cells. (**B**,**C**) The number of captured HCT116 cells and capture efficiency versus the number of seeded cells on the 50 μg/mL of anti-EpCAM-conjugated pCB hydrogel. *n* = 4.

The efficiency of CTC capture on the pCB hydrogels was first evaluated and were conjugated with different amounts of antibodies, from 1 μg Ab/mL to 80 μg Ab/mL. In the first experiment, the substrates captured cells from a solution containing 2000 HCT116 cells. The number of captured cells increased with increasing antibody concentrations (Figure 3A). A linear correlation between captured cells and antibody concentrations from ~700 cells (~35%) for 1 μg Ab/mL to ~1400 cells (~70%) for 20 μg Ab/mL. The capture efficiency reached 88% when the antibody concentration was 40 μg Ab/mL, while all the cells were captured on the pCB hydrogels conjugated with 80 μg Ab/mL. 

Next, the pCB hydrogel conjugated with 50 μg Ab/mL was used to evaluate the efficacy of cell capture from various cell numbers in solutions. When the number of cells was greater than 1000, less than 70% of the cells were captured, and the percentage of the captured cells decreased with increasing cell numbers (Figure 3B). The cell capture efficiency was only 69 and 42% when the number of cell seeding was about 1600 and 3300 cells, respectively. When the number of cells was less than 1000, the capture efficiency was greater than 90% and did not depend on the number of cells in the solution (Figure 3C).

### 2.3. Capture of HCT116 Cells from Blood 

**Figure 4:**
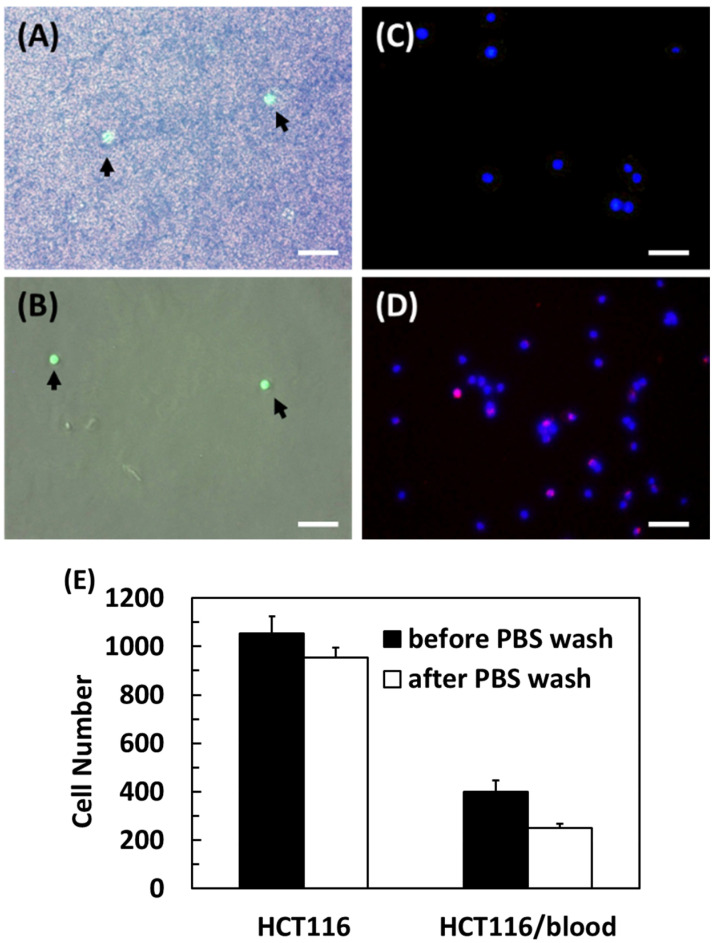
HCT116 cells (pre-stained by Calcein AM green fluorescence) were spiked in healthy human blood and incubated on the anti-EpCAM-conjugated pCB hydrogel for 4 h. The green fluorescent images were merged with the phase contrast images before (**A**) and after (**B**) PBS rinses. (**C**) The HCT116 cells captured on (C) the anti-EpCAM-conjugated pCB hydrogel, and (**D**) a glass slide were stained with DAPI for cell nucleus (blue) and anti-CD 45 for macrophages (red). (**E**) The cell numbers of HCT116 on anti-EpCAM-conjugated pCB hydrogel were counted before and after PBS rinses. Scale bar = 50 μm. *n* = 4. The arrows indicate HCT116 cells.

The efficacy of the anti-EpCAM antibody-conjugated pCB hydrogel for the capture of HCT116 cells from blood was next evaluated. When the hydrogel was incubated with the HCT116 cells/blood mixture, the surface was occupied with many blood cells and several HCT116 cells (indicated by arrows) before washing with PBS (Figure 4A, an image overlapped from a green fluorescent image and a phase contrast image). After rinsing with PBS, all blood cells were removed from the hydrogel, while HCT116 cells remained on the surface (Figure 4B), indicating that the antibody captured the HCT116 cells.

The efficiency of anti-EpCAM functionalized pCB hydrogels in the CTC capture from diluted human blood was next evaluated. A total of 1000 HCT116 cells were added to 1/8 diluted human blood and then encountered the anti-EpCAM functionalized hydrogel for 4 h. Compared to HCT116 cells alone with more than 90% capture efficiency, the capture efficiency of HCT116 cells spiked in human blood decreased to about 45% (Figure 4E). This might be because blood cells interfere with the interactions between HCT116 cells and surface anti-EpCAM moieties.

**Figure 5:**
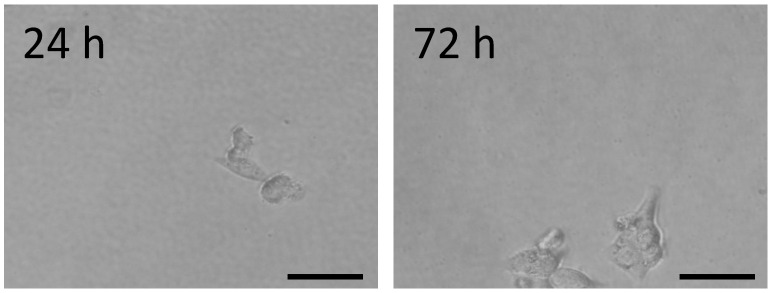
HCT116 cells captured were removed from the degradable anti-EpCAM pCB hydrogel and seeded on a tissue culture plate. Cell morphology was captured after being reseeded on a tissue culture plate for 24 and 72 h. Scale bar = 50 μm.

Several previous reports applied aptamer functionalized surfaces, anti-body-conjugated phosphocholine bilayer devices, geometric chips for CTC capture, or microfibers immobilized with enzyme-cleavable peptide [6,10,28,29]. To release the captured tumor cells, these studies used restriction endonuclease to cut the aptamer, EDTA to disrupt the phosphocholine bilayer, or enzymes to digest ECM proteins. These treatments raise concerns about cell damage. Here, we proposed a mechanism to harvest captured cells via the breakage of disulfide bonds in the crosslinkers via the addition of a natural amino acid, cysteine. As the hydrogel was dissociated by incubation with a 2 mM cysteine solution for 30 min, the captured cells detached from the hydrogels. The viability of the released cells was 94.78 ± 4.44%. The detached cells were able to attach and spread on a tissue culture plate (Figure 5). The results indicate that the release process is mild for the cells.

More recently, microfluidic-based CTC chips have been developed for the detection and enrichment of CTCs [10,30,31,32,33]. The flow rate in the microfluidic system prevents the attachment of blood cells to the surface to vastly increase the sensitivity and yield of the capture of rare cell populations from whole blood. Additionally, the device offers the ability to use very small quantities of samples and reagents to carry out separations and detections. Taking this step further, a microfluidic device based on the anti-EpCAM pCB hydrogel could be developed to provide the detection and enrichment of CTCs from blood cells in the future.

## 3. Conclusions

In summary, a platform for the CTC capture and release based on a non-fouling pCB hydrogel was demonstrated. The pCB hydrogel with disulfide-contained crosslinkers was successfully manufactured to inhibit the adhesion of HCT116 cells and blood cells. The pCB hydrogel with carboxyl groups was able to conjugate with anti-EpCAM via a carbodiimide reaction to specifically bind HCT116 cells in human blood, and demonstrated a capture efficiency as high as 45%. Furthermore, the bound HCT116 cells could be recovered via an addition of cysteine with limited damage. These results demonstrate potential applications for rare cell detection, purification, and subsequent cell proliferation via the robust platform of the anti-EpCAM-functionalized pCB hydrogel.

## 4. Materials and Methods

### 4.1. Materials

Ammonium persulfate (APS), N,N,N’,N’-tetramethylethylenediamine (TEMED) were purchased from Sigma-Aldrich. 2-carboxy-*N*,*N*,-dimethyl-*N*-(2′-(methacryloyloxy)ethyl)- ethanaminium inner salt (carboxybetaine methacrylate, CBMA) and disulfide-containing crosslinker, N, N’-dimethacryloylcystine (NDMCC) were synthesized according to a previously published procedure [34,35].

Culture medium for HCT116 cells (a human colon cancer cell line) contained Dulbecco’s modified Eagle’s medium (DMEM; Invitrogen) supplemented with 10% FBS, 2 mM L-glutamine, 100 units/mL penicillin, and 10 μg/mL streptomycin. Phosphate buffered saline (PBS) was prepared with 137 mM NaCl, 2.7 mM KCl, 10 mM Na_2_HPO_4_, and 1.8 mM KH_2_PO_4_, pH 7.4. 2-(*N*-morpholino) ethanesulfonic acid (MES) buffer was composed of 100 mM NaCl and 10 mM MES, pH 5.5, while *N*-Cyclohexyl-2-aminoethanesulfonic acid (CHES) buffer was composed of 10 mM NaCl and 50 mM CHES, pH 9. The hydrogel dissociation buffer contained 5 mM L-cysteine in PBS.

### 4.2. Fabrication of Anti-EpCAM Antibody-Conjugated pCB Hydrogel

CBMA was dissolved in PBS to 40% (*w*/*v*). The crosslinker (NDMCC) and the initiator (APS/TEMED) were added to the CBMA solution at a concentration of 5 mol% of CBMA and 10 mM, respectively. The monomer solution was immediately coated on a glass coverslip deposited with poly(4-vinyl-p-xylylene-co-p-xylylene) [36] and kept at 37 °C for 30 min for hydrogel formation. The formed pCB hydrogel layer was soaked in PBS until use. The water content and compressive moduli of pCB hydrogels were approximately 95% and 4.5 MPa, respectively [25].

Monoclonal antibody against EpCAM was generated, as described previously. The pCB hydrogel was immersed in MES buffer containing 50 mM NHS and 100 mM EDC for 1 h at room temperature, and then incubated with anti-EpCAM antibodies (50 μg Ab/mL) in PBS for 2 h. The antibody-conjugated hydrogels were stored in PBS before use.

### 4.3. Capture of HCT116 Cells on an Anti-EpCAM-Conjugated Hydrogel

HCT116 cells were expanded in T75 flasks in a humidified incubator with 5% CO_2_ at 37 °C. Cells were stained with Calcein AM green (Invitrogen) for 30 min at 37 °C and then harvested by trypsin treatment. The cell numbers in suspension were determined using a hemocytometer. In human blood tests, which were approved by the IRB on Biomedical Science Research, Academia Sinica (AS-IRB01-12106), blood samples from healthy donors were drawn and collected in 10 mL Vacutainer tubes containing the anticoagulant EDTA (BD Biosciences). When blood was used in the experiments, cells stained with Calcein AM green were spiked in diluted or whole blood. HCT116 cells were allowed to adhere to the antibody-conjugated pCB hydrogel at room temperature for 4 h. The cell numbers on the substrates were counted from microscopic images.

### 4.4. Statistical Analysis

Data were reported as means ± standard deviation (SD). Statistical analyses between different groups were determined using the Student’s *t* test. The probabilities of *p* < 0.05 were considered a significant difference. All statistical analyses were performed using the GraphPad Instat 3.0 program (GraphPad Software, La Jolla, CA, USA).

## Data Availability

Not applicable.

## Abbreviations

Ab	Antibody
APS	Ammonium persulfate
CBMA	Carboxybetaine methacrylate
CTCs	Circulating tumor cells
DAPI	4′,6-Diamidino-2-phenylindole
DMEM	Dulbecco’s modified Eagle’s medium
EDC	1-Ethyl-3-(3-dimethylaminopropyl)carbodiimide
EDTA	Ethylenediaminetetraacetic acid
EpCAM	Epithelial cell adhesion molecule
NDMCC	N, N’-dimethacryloylcystine
MES	2-(N-morpholino)ethanesulfonic acid
NHS	N-Hydroxysuccinimide
pCB	Poly(carboxybetaine methacrylate)
PBS	Phosphate-buffered saline
TEMED	N,N,N’,N’-tetramethylethylenediamine
